# Neural evidence for exercise-driven emotional stability via cognitive regulation mechanisms

**DOI:** 10.3389/fpsyg.2026.1775023

**Published:** 2026-03-19

**Authors:** Xuru Wang, Chenglin Zhou, Tianze Liu

**Affiliations:** 1 Shanghai Institute of Early Childhood Education, Shanghai Normal University, Shanghai, China; 2 School of Psychology, Shanghai University of Sport, Shanghai, China; 3 Department of Orthopedics, Changhai Hospital, Naval Medical University (Second Military Medical University), Shanghai, China

**Keywords:** acute aerobic exercise, cognitive reappraisal, emotion regulation, expressive suppression, fNIRS, prefrontal cortex

## Abstract

**Objective:**

This study investigated how acute aerobic exercise influences prefrontal cortex (PFC) activation and, in turn, modulates the implementation of two emotion regulation strategies—cognitive reappraisal and expressive suppression.

**Methods:**

Forty-three undergraduate students completed an emotion regulation task (ERT) before and after exercise under four conditions: viewing neutral images (WNeu), viewing negative images (WNeg), cognitive reappraisal (CR), and expressive suppression (ES). A subsequent recognition task was used to verify participants’ engagement in ERT. The exercise intervention consisted of 30 min of moderate-intensity cycling, preceded by a 5-min warm-up and followed by a 5-min cool-down. PFC activity was continuously recorded using functional near-infrared spectroscopy (fNIRS) during both exercise and the ERT.

**Results:**

Compared with the pre-exercise ERT session, participants’ negative affect during passive viewing of neutral or negative images remained unchanged. However, both cognitive reappraisal and expressive suppression showed significantly enhanced efficacy in reducing negative emotional experience after exercise. Notably, post-exercise reductions in right ventrolateral prefrontal cortex (rVLPFC) activation during expressive suppression were associated with greater decreases in negative affect. During exercise, prefrontal activation largely resembled resting levels, except for a decrease in left VLPFC activation, for which no significant association was observed with post-exercise regulation outcomes.

**Conclusion:**

The elicitation of negative emotion appears relatively stable, whereas a single session of moderate-intensity aerobic exercise enhances neural efficiency in the prefrontal cortex—improving emotion regulation performance while requiring fewer neural resources.

## 1 Introduction

Emotional regulation refers to the processes by which individuals influence the occurrence, experience, and expression of emotions (Gross, 1998). Effective emotional regulation, particularly through cognitive reappraisal and expressive suppression, plays a critical role in managing negative emotions (Gross and John, 2003). Cognitive reappraisal is an antecedent-focused strategy that reinterprets emotional events in a non-emotional manner (e.g., viewing a breakup as mutually beneficial), whereas expressive suppression is a response-focused strategy that modifies emotional responses by inhibiting related behaviors, such as facial expressions or bodily actions (Goldin et al., 2008). Both strategies have been shown to attenuate amygdala activity elicited by negative stimuli and depend on the recruitment of prefrontal resources, particularly the lateral prefrontal cortex (lPFC) (Bo et al., 2024; Sikka et al., 2022).

Aerobic exercise is linked to enhanced emotional well-being and reduced emotional disorders (Ströhle, 2009). While it does not alter the emotional situations individuals encounter, recent studies provide preliminary evidence that it can strengthen emotion regulation and thereby reduce the adverse effects of stress (Wang et al., 2024). Specifically, aerobic exercise modulates neural activity and functional dynamics in regions such as lPFC, which are also engaged during the use of cognitive reappraisal and expressive suppression strategies (Gao et al., 2022; Ge et al., 2021; Giles et al., 2014). According to the neural efficiency hypothesis, exercise enhances cortical efficiency in cognitive tasks, reflected in improved performance and reduced energy expenditure for equivalent outcomes, thereby facilitating more efficient cognitive processing (Audiffren, 2009). Given the central role of the prefrontal cortex in emotion processing and regulation (Dixon et al., 2017; Etkin et al., 2015), exercise-induced improvements in cognitive reappraisal and expressive suppression strategies are likely mediated by favorable activation patterns within this region.

Specifically, research on the potential effects of aerobic exercise on expressive suppression remains limited. However, as expressive suppression involves the general inhibition of overt behaviors, and aerobic exercise has been widely shown to enhance prefrontal functions supporting cognitive control (Byun et al., 2014; Yanagisawa et al., 2010), it is reasonable to hypothesize that aerobic exercise may also facilitate the regulatory effectiveness of the expressive suppression strategy. By contrast, a modest body of evidence indicates that aerobic exercise may promote cognitive reappraisal. Perchtold-Stefan et al. (2020) reported that individuals with higher levels of physical activity demonstrated greater use of reappraisal when facing adversity, employed more positive reinterpretations, and exhibited higher reappraisal quality. Similarly, Giles et al. (2017) found that physically active individuals achieved higher success rates in cognitive reappraisal tasks, and subsequent work showed that even a single bout of aerobic exercise can significantly enhance the effectiveness of reappraisal in reducing negative emotions (Giles et al., 2018).

Although behavioral findings support the beneficial effects of aerobic exercise on cognitive reappraisal, evidence from neuroimaging remains inconclusive. For instance, when functional near-infrared spectroscopy (fNIRS) and electroencephalography (EEG) were used to monitor prefrontal hemodynamic and electrophysiological activity before and after exercise, changes in prefrontal activation during emotional regulation did not reach statistical significance (Giles et al., 2018; Zhang et al., 2022). One possible explanation is that prefrontal oxygenation levels return rapidly to baseline after exercise (Fumoto et al., 2010), and the post-exercise assessments in these studies may have been conducted with relatively long delays, in some cases nearly 30 min (Zhang et al., 2022). Consistent with this view, a meta-analysis highlighted the importance of cognitive task timing relative to exercise cessation: cognitive tasks performed immediately or shortly after exercise showed stronger effects and better performance, whereas tasks administered 15 min or more after exercise exhibited diminished effects (Lambourne and Tomporowski, 2010). Another possible reason is related to the exercise modality used in some intervention studies, such as treadmill running (Giles et al., 2018). During running, body and head movements can introduce artifacts and compromise signal quality in the channels. When too many channels with low signal-to-noise ratio are present, not only is strict artifact correction required, but participants with excessive faulty channels must also be excluded from the analysis (Carius et al., 2023). Additionally, it is also possible that the facilitatory effect of exercise on emotion regulation through enhanced prefrontal cognitive processing occurs primarily during exercise itself. However, emotion-focused intervention studies have rarely monitored neural activity during the exercise period. Accordingly, the present study employed portable fNIRS to record prefrontal oxygenation not only before and after exercise during emotion regulation but also during exercise itself, thereby attempting to identify potential prefrontal neural substrates underlying exercise-induced improvements in cognitive reappraisal and expressive suppression.

In light of the above findings, the present study employed portable fNIRS to monitor prefrontal oxygenation during and after exercise, as this technique is relatively tolerant to motion artifacts. To further minimize movement-related noise, we adopted cycling on a power ergometer as the intervention, which allows the upper body to remain relatively stable and reduces head motion, thereby facilitating signal acquisition. Exercise intensity was set at a moderate level based on both physiological and affective considerations. Empirical evidence indicates an inverted U-shaped relationship between aerobic exercise intensity and emotional states: low-intensity exercise (e.g., walking below 40% HRmax) tends to be accompanied by stronger amygdala responses to negative stimuli (Chen et al., 2019), moderate intensity helps attenuate negative affect (Ge et al., 2021), whereas high-intensity exercise may elevate physiological stress and even induce negative emotions (Paolucci et al., 2018; Tempest and Parfitt, 2017). At the physiological level, moderate-intensity exercise is known to increase catecholamine release (e.g., dopamine), suppress serotonergic activity, elevate plasma endorphin concentrations to induce positive affect, and enhance endocannabinoid signaling to alleviate anxiety (Dinas et al., 2011; Greenwood, 2019; Mikkelsen et al., 2017).

Based on the neural efficiency hypothesis and prior empirical evidence, we hypothesized that moderate-intensity aerobic exercise would enhance prefrontal activation during exercise, thereby improving cognitive processing efficiency in this region and subsequently strengthening the regulatory effectiveness of cognitive reappraisal and expressive suppression without requiring additional cognitive resources. The findings of this study are expected to provide neuroimaging evidence for the role of aerobic exercise in improving negative emotion regulation and offer practical guidance for populations in need of enhanced emotion regulation capacity.

## 2 Materials and methods

### 2.1 Participants

Forty-three undergraduate students (18 males, 25 females, non-sports majors) participated voluntarily and received 150 RMB compensation. All were 18–25 years old, right-handed, with normal or corrected vision, and reported no history of emotional disorders, psychotropic medication, physical disability, or exercise contraindications. Participants did not engage in regular exercise outside compulsory physical education courses during the semester and met predefined criteria for low physical activity levels (<30 min per day and <2 exercise sessions per week) over at least the preceding 2 years; none had received formal athletic training. The study was approved by the local ethics committee (No.102772019RT044), and all participants provided written informed consent.

### 2.2 Questionnaires

To assess participant characteristics, the International Physical Activity Questionnaire–Short Form was used to measure physical activity levels (MET; higher scores indicate greater activity; Craig et al., 2003). Psychological assessments included the Beck Depression Inventory (Beck, 1961), the State–Trait Anxiety Inventory—Trait version (Spielberger et al., 1983), the Chinese version of the Positive and Negative Affect Schedule (Watson et al., 1988), and the Emotion Regulation Questionnaire (Gross and John, 2003). Results indicated that all participants were in a stable emotional state, with no significant sex differences in physical activity, depression, trait anxiety, affect, or habitual emotion regulation strategies. Demographic and psychological details are shown in Table 1.

**Table 1:** Demographic and psychological characteristics of participants.

Variables	Male (*n* = 18)	Female (*n* = 25)	*t*(41)	*p*
Age (y)	20.11 ± 0.46	20.20 ± 0.42	−0.14	0.89
Height (m)	1.75 ± 0.02	1.62 ± 0.01	7.01	<0.001***
Weight (kg)	63.97 ± 1.64	52.88 ± 1.02	6.06	<0.001***
BMI (kg/m^2^)	20.91 ± 0.33	20.17 ± 0.28	1.70	0.10
MET	2671.36 ± 384.85	1627.28 ± 363.78	1.94	0.06
HRrest	71.28 ± 2.10	73.80 ± 1.90	−0.88	0.38
BDI	2.56 ± 0.72	3.32 ± 0.76	−0.71	0.48
STAI-T	37.78 ± 1.85	39.60 ± 1.55	−0.76	0.45
PANAS				
Positive affect	29.78 ± 1.72	26.64 ± 1.64	1.29	0.20
Negative affect	13.61 ± 1.06	12.88 ± 0.70	0.60	0.55
ERQ				
Reappraisal	32.61 ± 1.41	31.16 ± 1.15	0.80	0.43
Suppression	14.94 ± 1.24	15.84 ± 1.21	−0.51	0.62

Values are presented as *M* ± *SE*. Independent-samples *t*-tests were conducted to compare male and female participants. BMI, body mass index; MET, metabolic equivalent of task; BDI, Beck Depression Inventory; PANAS, Positive and Negative Affect Schedule; ERQ, Emotion Regulation Questionnaire. ***p < 0.001.

### 2.3 Emotional regulation task

#### 2.3.1 Stimuli

A total of 240 pictures were selected from the International Affective Picture System (IAPS; Lang et al., 1999), consisting of two sets of neutral images (Neu1–2) and six sets of negative images (Neg1–6), with 30 pictures per set. Pictures were matched on valence and arousal within the IAPS, ensuring comparability across the six negative sets and the two neutral sets. Because cultural differences may influence emotional responses, 20 additional raters (10 males, 10 females, age = 19.65 ± 1.60) meeting the same inclusion criteria were recruited to provide ratings tailored to the Chinese sample. The raters evaluated each picture’s valence and arousal using the IAPS 9-point Likert scale (1 = low, 9 = high).

**Figure 1:**
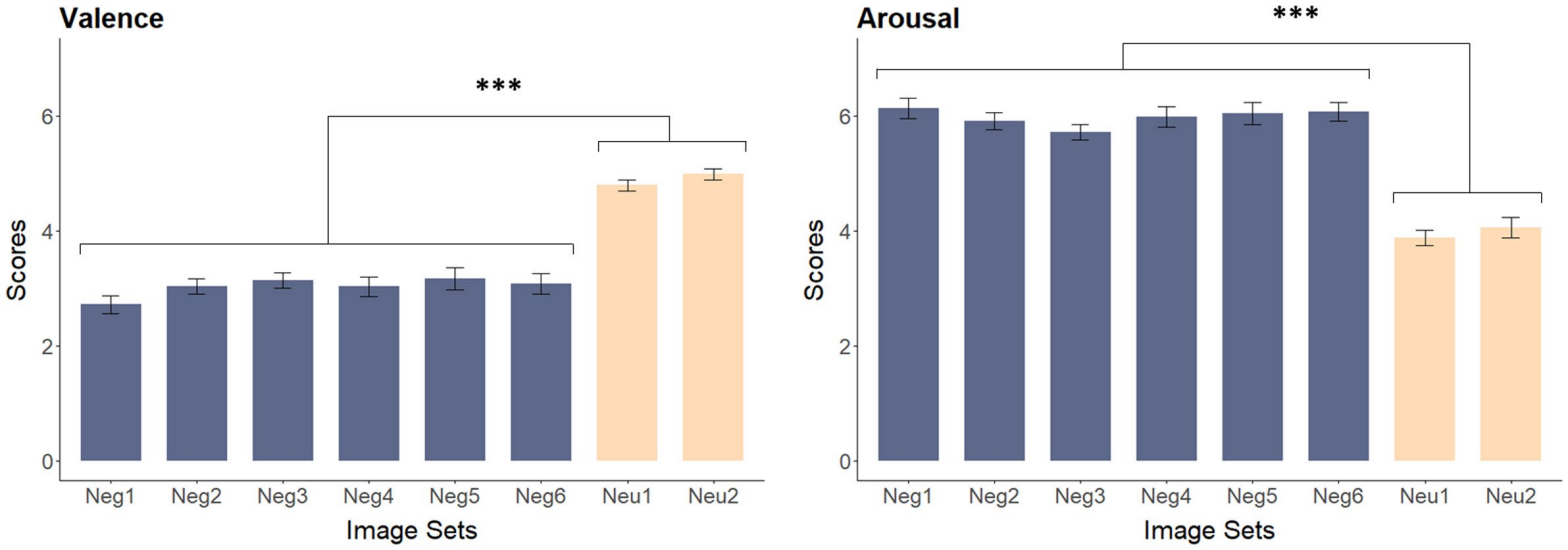
Comparison of valence and arousal rating results across image sets (Mean ± SE). ***p < 0.001.

One-way ANOVAs on the eight sets of images revealed significant effects of valence, *F*(7, 232) = 36.12, *p* < 0.001, and arousal, *F*(7, 232) = 32.13, *p* < 0.001. Bonferroni *post hoc* tests confirmed significant differences between neutral and negative sets on both valence (*p* < 0.001) and arousal (*p* < 0.001), no differences between the two neutral sets or across the six negative sets (*p*s > 0.82; see Figure 1). Overall, the six negative sets reliably elicited negative emotions and were comparable in affective properties.

#### 2.3.2 Experimental task

The task employed a block-design picture-viewing paradigm consisting of four blocks, each representing one condition: viewing neutral images (WNeu), viewing negative images (WNeg), cognitive reappraisal (CR), and expressive suppression (ES). The WNeu block consisted of neutral pictures and served as the non-emotional baseline, whereas the WNeg, CR, and ES blocks each used negative pictures with participants followed distinct instructions. In WNeu and WNeg, they passively viewed the pictures. In CR, they reappraised the images—for instance by questioning their realism or imagining positive outcomes—while avoiding expression control or attention shifts. In ES, they suppressed outward expressions and bodily reactions without altering interpretation or diverting attention.

**Figure 2:**
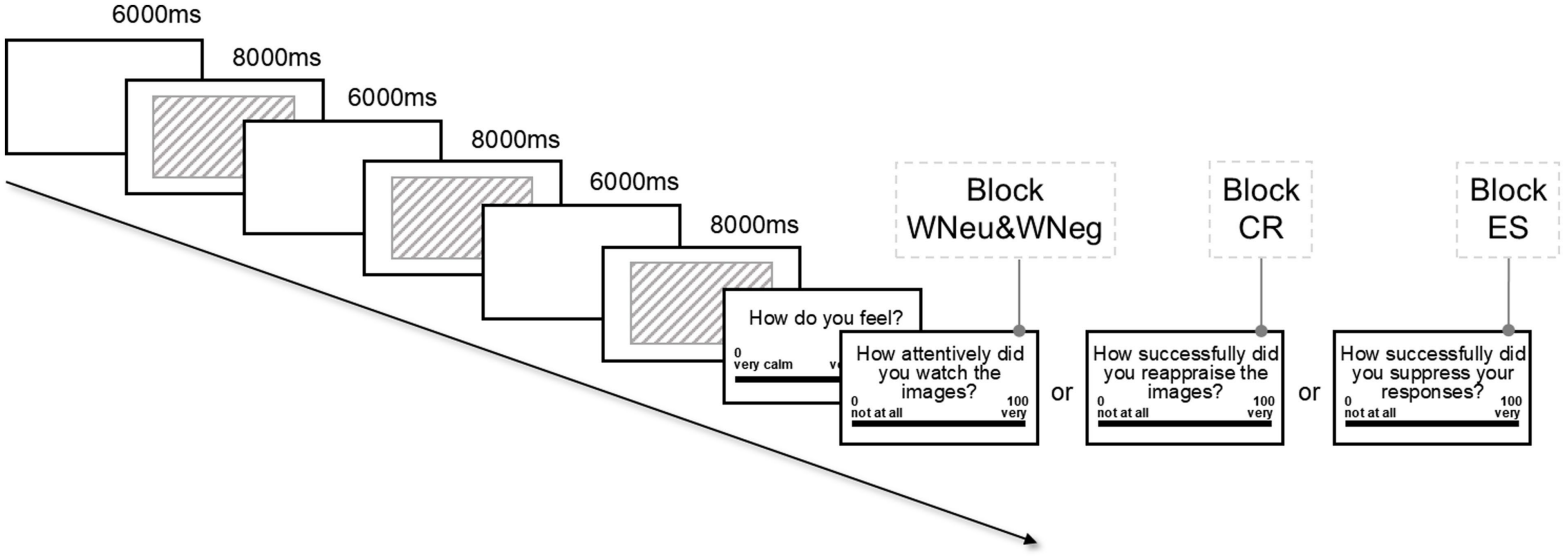
The flow chart of emotion regulation task.

Before each block, participants read task-specific instructions and a prompt (“Please watch carefully,” “Please reappraise,” or “Please suppress expressions”) and initiated the trials by pressing a key. Blocks contained 30 trials, with pictures shown for 8,000 ms and separated by a 6,000 ms interval. After every third trial, participants rated their emotional state on a 0–100 visual analogue scale (VAS; 0 = very calm, 100 = very negative) and completed a manipulation check of instruction compliance (attentive viewing, successful reappraisal, or suppression; 0 = not at all, 100 = very), by clicking on a line displayed on the screen. A two-minute break separated blocks to restore baseline affect. Blocks were presented in a fixed order (WNeu→WNeg→CR→ES) to avoid carryover effects on the neutral condition (Gross, 2002). As prior work shows no order effects for watch, reappraise, or suppress conditions (Goldin et al., 2008), these were not counterbalanced. The flow chart of emotion regulation task is presented in Figure 2.

The task was administered twice, once before the exercise intervention (ERT-pre) and once after (ERT-post). To minimize habituation, different but affectively matched pictures were used across runs. Prior to the formal experiment, participants received detailed instructions and completed practice trials with non-overlapping pictures.

### 2.4 Picture recall task

#### 2.4.1 Stimuli

The recognition task included four sets of pictures. Two sets of old images (oldA and oldB) were drawn from ERT-pre and ERT-post, with 24 pictures each (six from each condition). Two sets of new images (newA and newB) comprised 6 neutral and 18 negative pictures each, selected from the IAPS but not previously shown in the emotional regulation task (ERT).

#### 2.4.2 Experimental task

As the ERT imposed no accuracy or reaction time requirements, participants might have avoided negative images by closing their eyes or looking away; therefore, a picture recall task was included to verify that they had carefully observed the stimuli. The task was administered twice, following the ERT conducted before and after the exercise intervention. Each version comprised 48 trials, with pictures randomly drawn from one old set and one new set (PRT-pre: oldA and newA; PRT-post: oldB and newB). On each trial, participants indicated within 6,000 ms whether the picture had appeared in the ERT by pressing Q (“appeared”) or P (“not appeared”). After each response, a fixation cross was presented for 500 ms before the next trial (For a schematic of trial structure, see Supplementary material S1). Before the formal task, participants completed eight practice trials (half old, half new); these practice pictures were not included in the main task.

### 2.5 Functional near-infrared spectroscopy

fNIRS data were collected using the NIRSport2 system (NIRx) with an optode layout of 8 sources (wavelengths: 760 and 850 nm) and 7 detectors, forming 20 channels over the prefrontal cortex. The optodes were mounted on an EASY-CAP with spacers (NIRSount NIRSport AG.0053B) to fix the source–detector distance at 3 cm. Sources and detectors were positioned according to the international 10–20 EEG system, with sources S5 and S4 located at FPz and Fz, and detector D4 at AFz. Channel-to-region correspondence with six subregions of the prefrontal cortex was determined based on Gao et al. (2022), Okamoto et al. (2004), and Tsuzuki et al. (2007), as shown in Table 2. Signals were recorded with Aurora software at a sampling rate of 8.7 Hz, and signal quality—assessed using the coefficient of variation—was consistently within the acceptable to excellent range, ensuring reliable data acquisition.

**Table 2:** fNIRS probe placement.

Prefrontal region	Hemisphere	Channel(s)
Frontopolar prefrontal cortex (FPA)	–	6, 12, 16
Orbitofrontal cortex (OFC)	–	11, 13
Ventrolateral prefrontal cortex (VLPFC)	Left	1, 3, 4
	Right	18, 19, 20
Dorsolateral prefrontal cortex (DLPFC)	Left	2, 5, 7, 8
	Right	10, 14, 15, 17

“–” indicates regions with too few channels to distinguish hemispheres.

### 2.6 Exercise protocol

The acute moderate-intensity aerobic exercise was performed on a cycle ergometer (MONARK Ergomedic 828E) at 60–70% of maximal heart rate (HRmax, estimated as 220 – age), with heart rate continuously recorded using a Polar H10 chest strap. Participants first completed a 5-min warm-up at low resistance. During the warm-up, participants gradually increased their cadence to 60–70 revolutions per minute (rpm) and maintained it, while resistance was progressively adjusted until they reached the target heart rate zone. They subsequently cycled for 30 min at this resistance while maintaining both cadence (60–70 rpm) and heart rate within the target range (129.39 ± 1.02 bpm; intensity 64.72 ± 5.06%). The exercise session was divided into six 5-min periods. During the final minute of each 5-min period, cued by a 14-inch laptop screen switching from a white to a black background, participants were instructed to keep their core stable, minimize head movement, and focus solely on cycling without drinking water or other task-irrelevant actions. After each 5-min period, participants reported their perceived exertion using the Borg scale (6–20; 6 = no exertion, 20 = maximal exertion), with an average rating of 12.62 ± 0.25. The intervention concluded with a 5-min cool-down, during which resistance was reduced to about half of the working load. To minimize potential confounds such as dehydration or external distractions, participants were provided with water and were not allowed to use electronic devices or listen to music.

### 2.7 Procedure

Participants visited the laboratory once. They were instructed to refrain from moderate-to-vigorous exercise, caffeine, and alcohol for 24 h prior to the session. Upon arrival, participants were fully debriefed and provided informed consent before being fitted with a Polar heart rate monitor. They then completed questionnaires while donning the fNIRS, and signal quality was adjusted.

**Figure 3:**
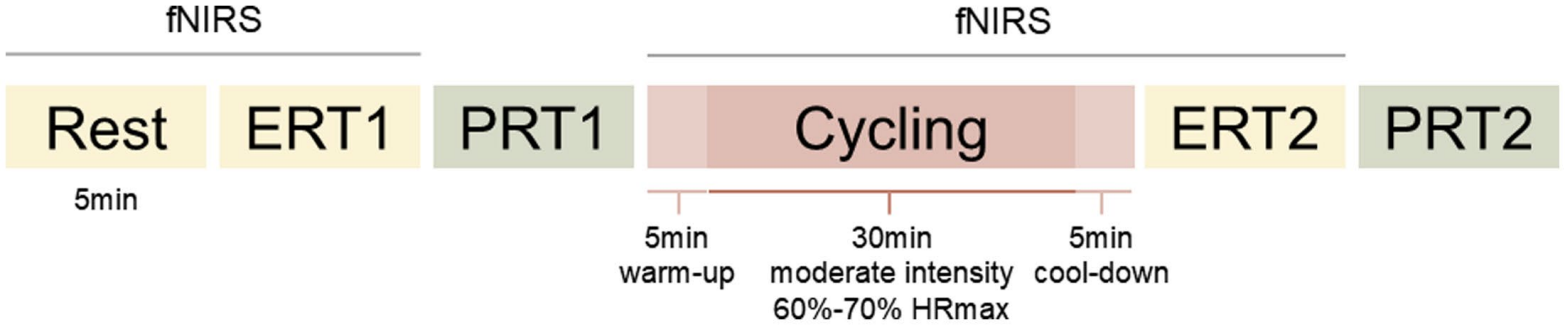
Schematic representation of the study session.

The session began with a 5-min eyes-open resting baseline, followed by a pre-exercise set of the ERT and its corresponding picture recall task (PRT). Participants then performed the aerobic exercise intervention, after which they completed a post-exercise ERT and PRT. Pre- and post-exercise ERTs employed two different stimulus sets, with set assignment pseudorandomized across participants. The interval between the exercise and the post-test was approximately 5 min. fNIRS signals were recorded during rest, throughout the exercise, and during each ERT. A schematic of the study session is presented in Figure 3.

### 2.8 Statistical methods

#### 2.8.1 Behavioral data

For the PRT, recognition accuracy was calculated for all participants, and those with accuracy below chance level were excluded.

For the ERT, unpleasantness ratings and manipulation check scores were analyzed using repeated-measures ANOVA with Time (pre-exercise, post-exercise) and Condition (WNeu, WNeg, CR, ES) as within-subject factors. Comparisons between WNeu and WNeg, as well as their interaction with Time, were used to verify whether the negative stimuli successfully induced negative affect and whether this effect was influenced by acute exercise. Comparisons of WNeg with CR and ES, and their interactions with Time, tested the regulatory effects of these two emotional regulation strategies and whether these effects were moderated by exercise.

When ANOVAs yielded significant interaction effects, *post hoc* tests with Bonferroni correction were performed. Effect sizes are reported as partial eta squared ( $\eta_{p}^{2}$ ) for ANOVAs and Cohen’s *d* for *t*-tests. When the assumption of sphericity was violated, Greenhouse–Geisser–corrected *p*-values were reported (corrected degrees of freedom rounded to integers). All analyses were conducted in R (version 4.5.1) using the bruceR package (Bao, 2024).

#### 2.8.2 fNIRS data

##### 2.8.2.1 ERT

Raw fNIRS signals were preprocessed using the Homer2 toolbox (version 2.0, Martinos Center for Biomedical Imaging) in MATLAB (2013b). Channels with extreme intensity or a signal-to-noise ratio <2 were excluded, and the remaining optical intensity signals were converted to optical density. Motion artifacts were corrected using spline interpolation combined with a Savitzky–Golay filter. For each 1.5-s segment, if signal changes exceeded 5 SDs (*std thresh*) or 50 times the amplitude (*amp thresh*), a 2-s window around the artifact was marked and corrected. Trials with artifacts that could not be corrected were discarded. Low- and high-pass filters were not applied, as subsequent GLM analyses included polynomial drift correction. Finally, cleaned optical density signals were converted to changes in oxygenated (HbO) and deoxygenated hemoglobin (HbR) using the hmrOD2Conc function (ppf = 6.0; Huppert et al., 2006) based on the modified Beer–Lambert law. According to neurovascular coupling, neuronal activation increases regional cerebral blood flow, leading to higher HbO and lower HbR; as HbO is more sensitive to experimental stimuli, analyses focused primarily on HbO (Pinti et al., 2020; Schaeffer et al., 2014; Strangman et al., 2002).

Preprocessed HbO time-series data were analyzed using a general linear model (GLM) implemented in SPM8 (Wellcome Department of Cognitive Neurology) and NIRS-SPM v4 (Bio Imaging Signal Processing Lab) running in MATLAB. A wavelet-MDL detrending algorithm and an hrf low-pass filter were applied to attenuate low-frequency drift, Mayer waves, respiration, and cardiac artifacts, ensuring that the signals primarily reflected cerebral hemodynamic responses. Task-related regressors were modeled using the onset and duration of picture stimuli, and GLM parameter estimation generated β weights for each channel and condition. For subsequent analyses, β weights were averaged across channels within six predefined prefrontal ROIs to obtain region-specific activation features.

HbO β weights for each ROI were analyzed using nonparametric permutation tests with Time (pre-exercise, post-exercise) and Condition (WNeu, WNeg, CR, ES) as within-subject factors. The number of permutations was set to 5,000. Because analyses were conducted separately for each ROI, false positives were controlled by applying FDR correction to main and interaction effects. Effect sizes are reported as partial eta squared ( $\eta_{p}^{2}$ ). To evaluate significant Time × Condition interactions, pairwise comparisons were conducted on the original data using the nonparametric Wilcoxon signed-rank test, with *post hoc* results corrected using FDR; for comparisons showing significant effects, medians (Mdn) were also reported.

For ROIs showing significant pre–post differences in a given condition, difference scores were calculated for brain activation (Δβ = β_post – β_pre) and behavioral unpleasantness ratings (ΔRating = Rating_post – Rating_pre). Pearson correlations between Δβ and ΔRating were then computed to assess brain–behavior associations. If significant correlations emerged, the corresponding ROI was used as a seed region to calculate functional connectivity with all other channels. Connectivity values were Fisher z-transformed and entered into permutation tests, with pairwise comparisons assessed using Wilcoxon signed-rank tests. When significant interaction effects were observed, Pearson correlations between changes in connectivity (Δz = z_post – z_pre) and changes in behavior (ΔRating) were further examined.

##### 2.8.2.2 Rest and exercise

Resting-state data were preprocessed by excluding low-quality channels, converting raw optical intensity to optical density, and correcting motion artifacts (parameters as in the ERT preprocessing). Signals were band-pass filtered (0.01–0.10 Hz) to remove physiological noise and low-frequency drift, and optical density was converted to oxygenated hemoglobin concentration ([HbO]). For each channel, [HbO] time series were averaged to obtain resting-state signals, and channel means within the six ROIs were further averaged to yield ROI-level [HbO] values for statistical analysis.

Exercise data were preprocessed in the same manner as resting-state data to obtain [HbO] signals. Warm-up and cool-down phases were excluded, retaining only the 30 min of moderate-intensity aerobic exercise. Data were segmented into 5-min periods, with the final 1 min of each period (i.e., minutes 4–5, 9–10, 14–15, 19–20, 24–25, and 29–30) averaged (illustration of time window selection, see Supplementary material S2), corresponding to intervals when participants were required to remain stable and refrain from unrelated activities such as drinking water. Channel-level [HbO] data were averaged and further aggregated across the six ROIs.

Paired Wilcoxon signed-rank tests were conducted to compare cycling [HbO] with resting [HbO], using rest as the baseline to assess activation across prefrontal regions during exercise. Effect sizes were reported as *r*, calculated as *r* (Z/ $\sqrt{N}$ ). For regions showing significant activation, difference scores were calculated (*Δ*[HbO] = Cycle – Rest), and Pearson correlations were performed between Δ[HbO] and ΔRating to examine whether changes in neural activity during cycling were associated with changes in emotional experience.

## 3 Results

### 3.1 Behavioral data

#### 3.1.1 PRT

**Figure 4:**
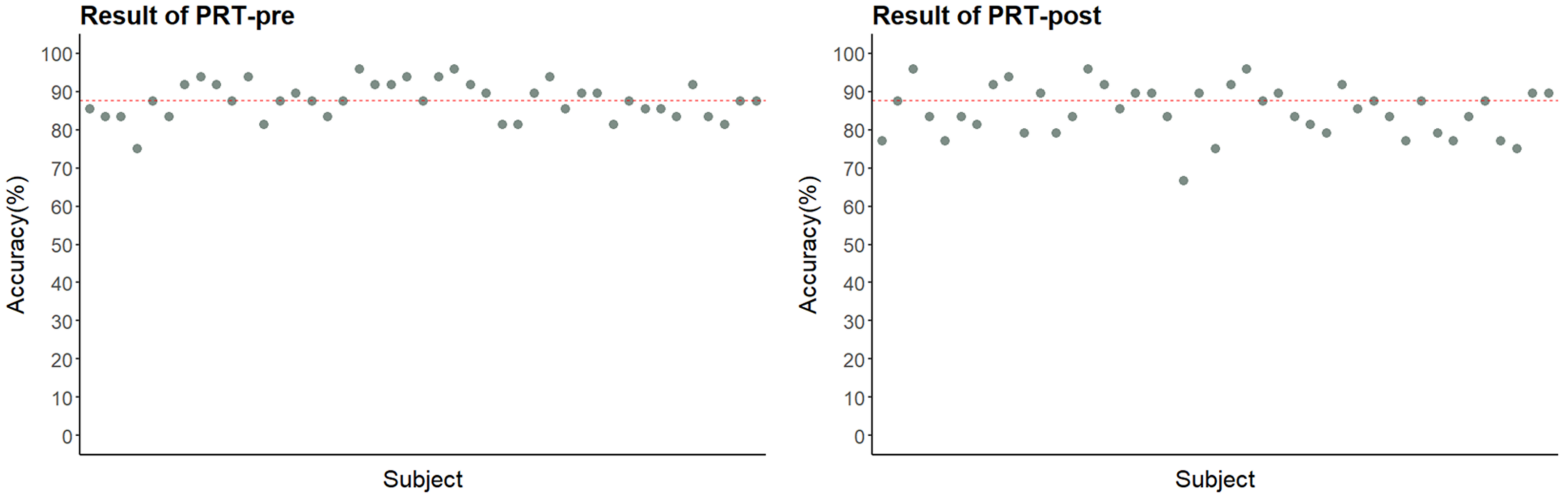
Accuracy of participants in the two PRT tests (pre- and post-exercise). The red dashed line indicates the mean accuracy.

Participants’ recognition accuracy was well above chance both before (*M* = 87.86%) and after exercise (*M* = 84.83%; *ps* < 0.001, Cohen’s *ds* > 5; Figure 4), indicating that no participants were excluded for lack of task engagement. A paired-samples *t*-test further showed that recognition accuracy for neutral pictures decreased significantly after exercise (pre = 90.65%, post = 84.99%; Cohen’s *d* = −0.54, *p* < 0.001), whereas accuracy for negative pictures did not change significantly (*p* = 0.06).

#### 3.1.2 ERT

##### 3.1.2.1 The unpleasant assessment

**Figure 5:**
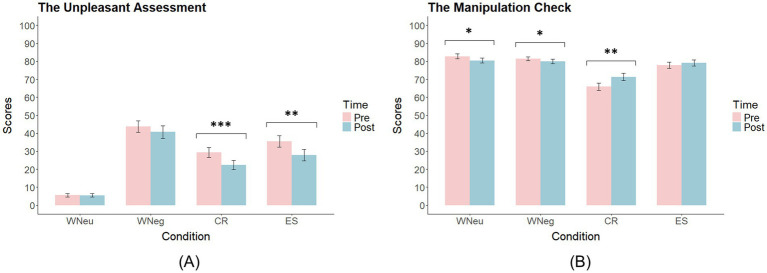
Ratings in the two ERT tests (pre- and post-exercise) (Mean ± SE). **(A)** The unpleasant assessment. **(B)** The manipulation check. *p < 0.05, **p < 0.01, ***p < 0.001.

Analysis of unpleasant ratings (0–100 VAS) revealed a significant main effect of Condition, *F*(2, 92) = 69.88, *p* < 0.001, $\eta_{p}^{2}$ = 0.63. *Post hoc* comparisons showed that unpleasant ratings were significantly higher in the WNeg condition (*M* = 42.22) than in the WNeu (*M* = 5.56), CR (*M* = 25.85), and ES (*M* = 31.62) conditions (all *ps* < 0.001). In addition, WNeu ratings were significantly lower than those in CR and ES (both *ps* < 0.001). A significant main effect of Time was also observed, *F*(1, 42) = 15.05, *p* < 0.001, $\eta_{p}^{2}$ = 0.26, with unpleasant ratings decreasing from pre- to post-exercise (Mpre = 28.51, Mpost = 24.11). Finally, the Condition × Time interaction was significant, *F*(2, 103) = 7.12, *p* = 0.017, $\eta_{p}^{2}$ = 0.09. Follow-up comparisons indicated that unpleasant ratings decreased significantly after exercise in the CR (Mpre = 29.29, Mpost = 22.41, *p* < 0.001) and ES (Mpre = 35.43, Mpost = 27.82, *p* = 0.005) conditions, whereas no pre–post changes were found in WNeu or WNeg (*ps* > 0.15) (see Figure 5A).

##### 3.1.2.2 The manipulation check

Analysis of conformation scores revealed no main effect of Time but a significant Condition × Time interaction, *F*(2, 87) = 7.76, *p* < 0.001, $\eta_{p}^{2}$ = 0.16. Simple effects analyses showed that after exercise, participants reported lower attentiveness when viewing neutral (Mpre = 82.79, Mpost = 80.49, *p* = 0.034) and negative pictures (Mpre = 81.54, Mpost = 79.99, *p* = 0.049). In contrast, the cognitive reappraisal strategy was reported as being used more successfully (Mpre = 65.98, Mpost = 71.33, *p* = 0.005). No pre–post differences were observed for expressive suppression success (*p* = 0.25) (see Figure 5B).

### 3.2 fNIRS data

#### 3.2.1 ERT

Five participants were excluded due to poor optode contact, and six more were discarded because excessive head movement during exercise led to low post-exercise signal quality. Consequently, data from 32 participants were included in the final analyses.

**Figure 6:**
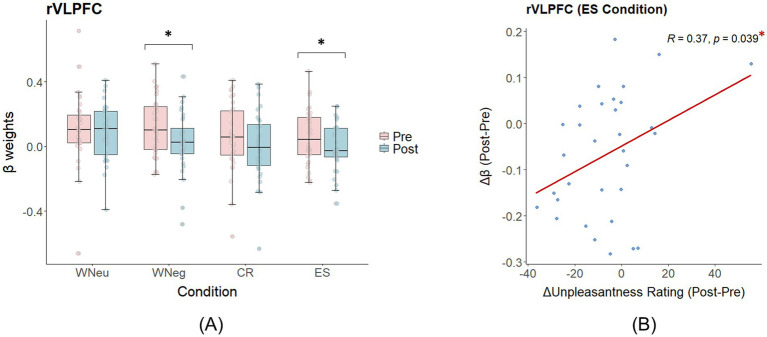
Main fNIRS results of the ERT (median ± interquartile range). **(A)** Changes in rVLPFC activation pre- and post-exercise. **(B)** Correlation between changes in rVLPFC activity and changes in unpleasantness ratings in the ES condition. *p < 0.05.

Permutation tests of HbO β weights across the six prefrontal ROIs revealed significant Condition × Time interactions in the right VLPFC (rVLPFC; *F* = 6.08, $\eta_{p}^{2}$ = 0.49, *p* = 0.013, *padj* = 0.0216), left VLPFC (lVLPFC; *F* = 5.48, $\eta_{p}^{2}$ = 0.66, *p* = 0.018, *padj* = 0.0216), OFC (*F* = 6.87, $\eta_{p}^{2}$ = 0.86, *p* = 0.0092, *padj* = 0.0216), FPA (*F* = 5.54, $\eta_{p}^{2}$ = 0.81, *p* = 0.0164, *padj* = 0.0216), and right DLPFC (rDLPFC; *F* = 7.81, $\eta_{p}^{2}$ = 0.90, *p* = 0.0042, *padj* = 0.0216). Wilcoxon signed-rank tests indicated no activation differences between WNeg and WNeu (emotional arousal) or between CR/ES and WNeg (emotional regulation) in either pre- or post-exercise sessions. The observed interactions were driven by reduced HbO β weights in specific conditions after exercise. Specifically, HbO β weights decreased from pre- to post-exercise in rVLPFC under WNeg (Mdn_pre_ = 0.10, Mdn_post_ = 0.03, *p* = 0.024) and ES (Mdn_pre_ = 0.04, Mdn_post_ = −0.03, *p* = 0.029) (see Figure 6A), in OFC under WNeu (Mdn_pre_ = 0.15, Mdn_post_ = 0.02, *p* = 0.033) and WNeg (Mdn_pre_ = 0.14, Mdn_post_ = −0.05, *p* = 0.024), in FPA under ES (Mdn_pre_ = 0.03, Mdn_post_ = 0.00, *p* = 0.029), and in rDLPFC under ES (Mdn_pre_ = 0.10, Mdn_post_ = 0.03, *p* = 0.028).

Pearson correlations between *Δ*β (βpost – βpre) and ΔRating (Ratingpost – Ratingpre) were then conducted for these regions. Only the rVLPFC in the ES condition showed a significant positive association (*r* = 0.37, *p* = 0.039), while all other correlations did not reach significance (*ps* > 0.20) (see Figure 6B).

**Figure 7:**
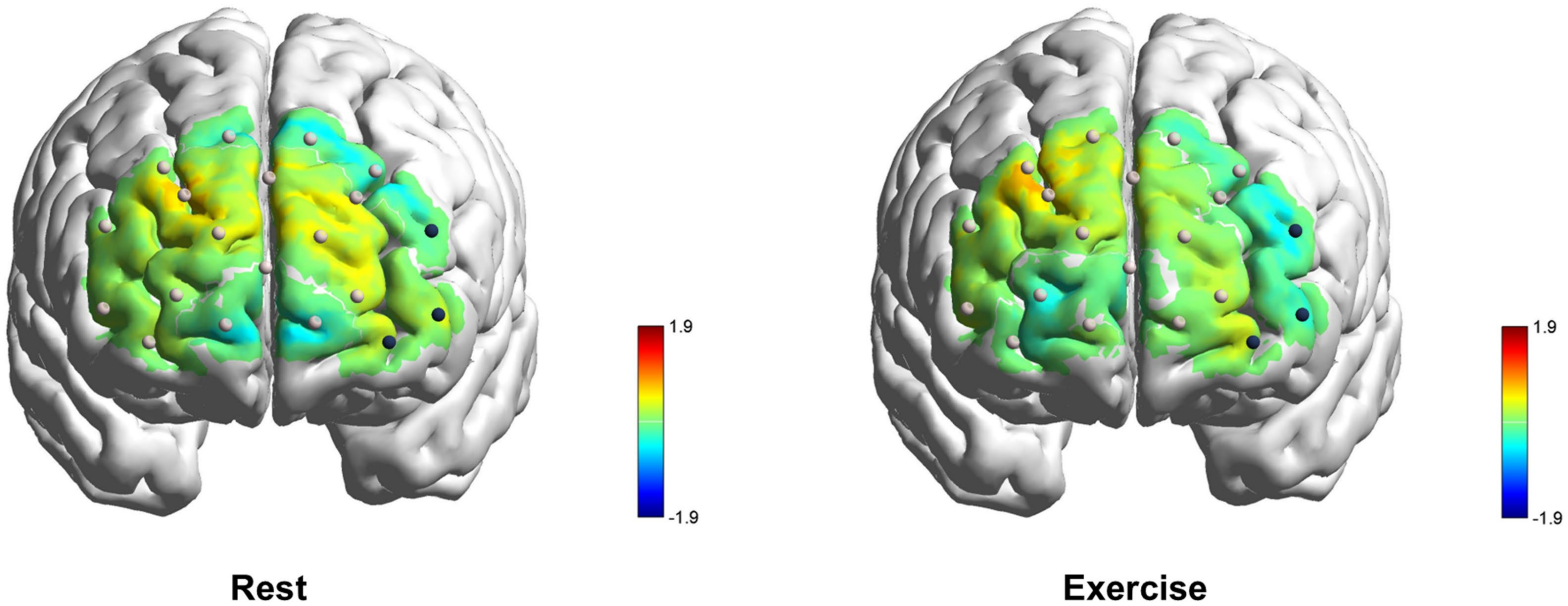
PFC activation across channels during rest and exercise. Colors represent *t*-values of mean [HbO] compared to zero. Solid spheres indicate the locations of measurement channels, with dark blue spheres denoting channels in lVLPFC that showed significant differences between the rest and exercise. The rendered brain images were generated using BrainNet Viewer (Xia et al., 2013).

Using the rVLPFC as a seed region, functional connectivity with the other five prefrontal ROIs was computed, Fisher z-transformed, and analyzed with permutation tests. Significant Condition × Time interactions emerged for the rVLPFC–lVLPFC (*F* = 4.94, $\eta_{p}^{2}$ = 0.99, *p* = 0.026, *padj* = 0.043), rVLPFC–rDLPFC (*F* = 14.71, $\eta_{p}^{2}$ = 0.95, *p* < 0.001, *padj* = 0.001), and rVLPFC–lDLPFC (*F* = 9.38, $\eta_{p}^{2}$ = 0.99, *p* = 0.002, *padj* = 0.004) pathways. Wilcoxon signed-rank tests showed no differences in z-values between WNeg and WNeu (emotional arousal) or between CR/ES and WNeg (emotional regulation) in either pre- or post-exercise sessions. The interactions were driven by enhanced post-exercise connectivity in the rVLPFC–rDLPFC pathway under WNeu (Mdn_pre_ = 0.33, Mdn_post_ = 0.45, *p* = 0.044) and WNeg (Mdn_pre_ = 0.33, Mdn_post_ = 0.47, *p* = 0.013), as well as in the rVLPFC–lDLPFC pathway under WNeu (Mdn_pre_ = 0.25, Mdn_post_ = 0.34, *p* = 0.044) and WNeg (Mdn_pre_ = 0.26, Mdn_post_ = 0.35, *p* = 0.008). Pearson correlations between connectivity changes (Δz = zpost – zpre) and changes in unpleasant ratings (ΔRating) did not reach significance (*p*s > 0.05) (Figure 7).

#### 3.2.2 Exercise intervention

Wilcoxon signed-rank tests on [HbO] across the six prefrontal ROIs revealed that, compared with rest, [HbO] in lVLPFC decreased during exercise (Mdn_rest_ = 4.20 × 10^−3^ μmol/L, Mdn_exercise_ = −17.69 × 10^−3^ μmol/L, *p* = 0.049, *r* = −0.35). However, Pearson correlation analyses showed no significant associations between Δ[HbO] in lVLPFC during exercise and changes in unpleasant ratings (ΔRating) under WNeu, WNeg, CR, or ES (all *ps* > 0.15).

## 4 Discussion

This study examined how acute moderate-intensity aerobic exercise affects cognitive reappraisal, expressive suppression, and associated changes in prefrontal oxygenation. The main findings demonstrated that aerobic exercise enhanced the efficacy of both cognitive reappraisal and expressive suppression in reducing negative emotional experience. In addition, it improved rVLPFC neural efficiency during expressive suppression, enabling greater regulation of negative affect with fewer recruited resources. Furthermore, prefrontal activation during exercise remained largely stable, with the exception of a decrease in the lVLPFC that was not significantly associated with emotion regulation outcomes.

In the ERT task, unpleasantness ratings confirmed that the experimental stimuli effectively elicited negative emotion, as participants reported greater unpleasantness when viewing negative compared with neutral pictures. Importantly, emotional experience during passive viewing of negative pictures did not differ between pre- and post-exercise sessions. This aligns with Tartar et al. (2018), who also found no pre–post aerobic exercise (30 min) differences in valence ratings of negative images, and with Ligeza et al. (2023), who reported that acute exercise may modulate brain responses, specifically the late positive potential (LPP) component of event-related potentials, to positive but not negative stimuli. Collectively, these findings suggest that baseline sensitivity to negative emotion represents a relatively stable trait that is unlikely to be modified by a single session of aerobic exercise.

Building on this stable baseline, both cognitive reappraisal and expressive suppression successfully downregulated negative emotional experience, with the magnitude of this reduction being significantly greater after exercise. This suggests that aerobic exercise amplify the effectiveness of emotion regulation in mitigating unpleasant affect. Our findings replicate prior work showing that a single session of treadmill running enhanced the efficacy of cognitive reappraisal in reducing negative emotion (Giles et al., 2018). Moreover, the manipulation check results converged with the unpleasantness ratings, as participants subjectively reported greater success in applying reappraisal after exercise. Although expressive suppression is mainly a response-focused strategy, prior research has shown that it can be effective in reducing negative affect (Goldin et al., 2008; Liverant et al., 2008). Given that expressive suppression tends to be more effective in alleviating negative emotion in the short term (Cai et al., 2016; Yuan et al., 2015), the present study required participants to report their emotions in real time rather than retrospectively (e.g., after completing an entire block). This design provided a more sensitive assessment of the regulatory effects of suppression and further demonstrated that its benefits can be amplified by aerobic exercise.

Our findings suggest that the beneficial effects of habitual exercise on emotion regulation partly rely on prefrontal mechanisms. Following acute exercise, rVLPFC activation during expressive suppression decreased, and this reduction was accompanied by greater improvements in downregulating negative affect. Prior studies consistently indicate that the lateral prefrontal cortex is engaged in both cognitive reappraisal and expressive suppression (Kohn et al., 2014; Morawetz et al., 2017), with a systematic review reporting that most investigations of suppression-related neural activity have observed VLPFC involvement (Sikka et al., 2022). Moreover, a tDCS study demonstrated a causal role of the rVLPFC in the downregulation of emotional responses (He et al., 2018). The observed post-exercise reduction in rVLPFC activation aligns with the neural efficiency hypothesis, whereby better task performance is achieved with fewer neural resources. Study in endurance-trained cyclists have shown enhanced neural efficiency in individuals with high VO₂max (Ludyga et al., 2016). Extending this evidence, our results indicate that such efficiency gains can also emerge after a single bout of exercise, not only in professional athletes with long-term training experience.

Consistent with the findings of Giles et al. (2018), we did not observe changes in lateral prefrontal activation during cognitive reappraisal following exercise. One possible explanation is that the efficacy of reappraisal may depend more on functional connectivity between PFC and amygdala than on PFC activation alone. Clinical studies have shown that patients with affective disorders exhibit greater prefrontal activation during cognitive reappraisal compared with healthy controls, yet demonstrate poorer regulatory outcomes and reduced amygdala downregulation (Kanske et al., 2012). Neuroimaging evidence further indicates that this population shows compromised integrity of the uncinate fasciculus connecting the prefrontal cortex and amygdala (Zheng et al., 2018). Supporting this view, a TMS study demonstrated that stimulation of the VLPFC modulated amygdala responses and, using fMRI, also identified a structural pathway linking the VlPFC and amygdala (Sydnor et al., 2022). The strength of negative functional connectivity within this pathway has also been shown to predict the effectiveness of reappraisal (Townsend et al., 2013). Together, these findings suggest that reappraisal efficacy may be better indexed by PFC–amygdala coupling than by PFC activation alone. Given that fNIRS is limited to cortical hemodynamics, future studies using imaging modalities that can assess subcortical structures (e.g., fMRI) may help to further examine exercise-related changes in reappraisal circuitry, particularly PFC–amygdala connectivity.

Contrary to our hypothesis, prefrontal activation remained relatively stable during exercise. Rather than an increase, a reduction in lVLPFC activation was observed. This may be attributable to both participant characteristics and methodological factors. First, neural activity during exercise is constrained by individual fitness levels. Previous work suggests an inverted U-shaped association between aerobic exercise intensity and prefrontal activation, with prefrontal oxygenation increasing at lower intensities but declining once intensity exceeds an individual-specific turning point (Wang et al., 2024). Notably, this turning point may vary with training status, occurring at higher intensities in trained individuals and at lower intensities in untrained individuals (Rooks et al., 2010). In untrained participants, greater engagement of motor networks (e.g., primary motor cortex and basal ganglia) may divert cerebral blood flow toward motor regions, reducing prefrontal oxygenation (Dietrich, 2006). In addition, moderate-to-high intensity exercise may induce hyperventilation in low-fitness individuals, leading to reduced arterial CO₂ (PaCO₂), cerebral arteriolar vasoconstriction, and consequently lower cortical oxygenation (Matta Mello Portugal et al., 2013). Given that our sample consisted of untrained participants with low habitual physical activity, the observed reduction in lVLPFC activation during exercise is therefore plausible. Second, our results differ from those of Gao et al. (2022), who reported increased prefrontal activation when analyzing a 25-min window. In contrast, we analyzed 1-min epochs at the end of each 5-min period, during which participants were instructed to remain still and focus solely on cycling. Gao’s approach may have captured unrelated activities such as drinking or task-unrelated thoughts, potentially inducing additional prefrontal responses.

## 5 Limitations

In the emotion regulation task, the order of blocks was fixed based on prior studies reporting minimal order effects (Goldin et al., 2008; Gross, 2002). However, because participant characteristics may vary across studies, a fixed sequence may still permit some inter-condition interference in our sample. In addition, no fixed time limit was imposed for the rating responses. Response time was not a primary outcome of interest; therefore, we did not impose a fixed time limit and allowed participants to report their subjective experience in a natural manner, with each rating response advancing automatically to the next trial. To support task engagement under this self-paced design, we included a subsequent picture-recognition task. Nevertheless, allowing self-paced ratings may introduce additional effects related to variability in rating duration. Future studies could test potential order effects across conditions in young, physically inactive adults using counterbalanced or randomized block orders, and use pilot response-time data to guide the selection of an appropriate rating window (or upper limit).

We employed only moderate-intensity aerobic exercise as the intervention. This choice was based on evidence suggesting an inverted-U relationship between exercise intensity and cognitive performance (Kamijo et al., 2007; Wang et al., 2024). In addition, we did not observe a direct association between neural activity during exercise and changes in emotion regulation outcomes, suggesting that PFC-related modulation of emotion may primarily emerge in the post-exercise regulation phase. Alternatively, this null finding may also reflect the relatively narrow intensity range (60–70%HRmax) employed in the present study. Future research should include a broader range of exercise intensities and integrate real-time monitoring of both respiratory (Tempest and Parfitt, 2017) and neural activity to better delineate the thresholds at which exercise enhances or impairs emotion regulation and to clarify the contribution of the prefrontal cortex. Additionally, HRmax was estimated using the age-predicted formula (220 − age) in this study. Although direct maximal exercise testing (e.g., incremental cycling to volitional exhaustion) can provide a more accurate assessment of HRmax (and VO₂max), we considered the safety and feasibility of such testing in our physically inactive sample. In future work—particularly with trained or habitually active cohorts—direct assessment of HRmax (and, where feasible, VO₂max) would allow more precise prescription of exercise intensity and intervention dosage.

To monitor neural activity during exercise, we employed fNIRS, a technique relatively tolerant to motion artifacts. However, fNIRS cannot capture activity in subcortical structures, preventing us from assessing the effects of acute aerobic exercise on classic emotion-regulation pathways such as the prefrontal–amygdala pathway. Moreover, increased scalp blood flow during exercise may have confounded the measurement of cortical oxygenation (Miyazawa et al., 2013), as the current device did not support short source–detector separation to distinguish scalp from cortical signals. Although our results indicated decreases rather than increases in prefrontal oxygenation during and after exercise, future studies could address this limitation by using devices with short-separation channels to better account for extracerebral blood flow (Herold et al., 2018). In addition, 32 participants were included in the fNIRS analysis. Several datasets were excluded due to low signal-to-noise ratios caused by excessive head/upper-limb motion during exercise, which is a challenge when collecting fNIRS data in exercise settings (Carius et al., 2023). This reduction in sample size may have limited statistical power and may have contributed to null findings (e.g., correlations between improvements in emotion-regulation efficacy and prefrontal activity). Future studies should therefore plan for a higher attrition rate at the design stage and recruit a larger initial sample to compensate for motion-related data loss.

## 6 Conclusion

The present study demonstrates that 30 min of moderate-intensity cycling exercise (excluding warm-up and cool-down) enhanced the effectiveness of both cognitive reappraisal and expressive suppression in regulating negative emotion, while leaving the generation of negative affect unchanged. Moreover, post-exercise reductions in rVLPFC activation during expressive suppression were positively associated with greater reductions in negative emotion, suggesting enhanced neural efficiency in this region. These findings add to the growing evidence on the intricate relationships among aerobic exercise, emotion, and emotion regulation, emphasizing the importance of exploring how variations in exercise load may further modulate emotion regulation and their underlying neural mechanisms.

## Data Availability

The raw data supporting the conclusions of this article will be made available by the authors, without undue reservation.
