# Supplementary material for: Neural evidence for exercise-driven emotional stability via cognitive regulation mechanisms
Source: Front Psychol. 2026 Mar 19;17:1775023. doi: 10.3389/fpsyg.2026.1775023 (PMC13044044; doi:10.3389/fpsyg.2026.1775023)
Supplement: Supplementary file 1 [file Supplementary_file_1.docx]

**S1**


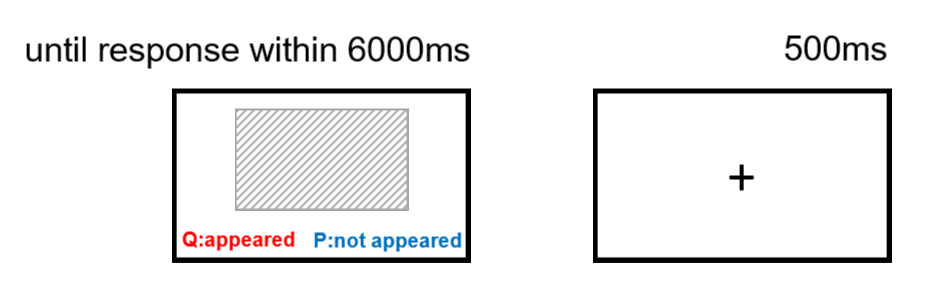


The flow chart of picture recognition task

**S2**
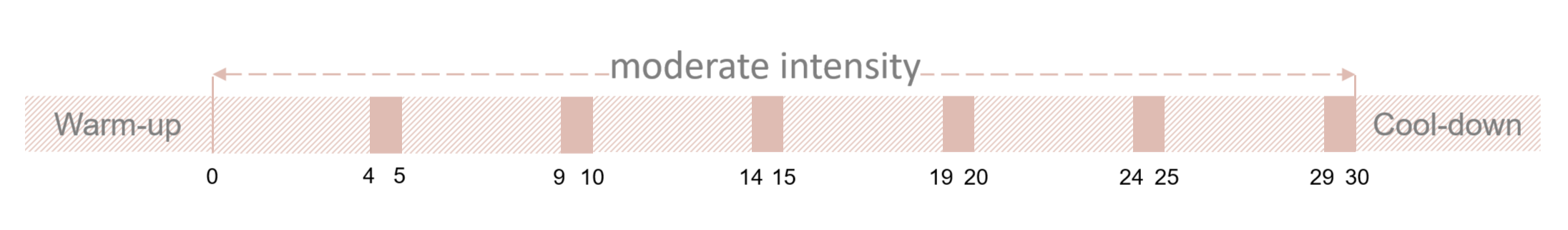


Selection of time windows for fNIRS data during the exercise session. The solid-colored segments denote the time intervals included in the analysis, whereas the hatched areas indicate the warm-up and cool-down phases that were excluded.
